# Differential Regulation of Human Aortic Smooth Muscle Cell Proliferation by Monocyte-Derived Macrophages from Diabetic Patients

**DOI:** 10.1371/journal.pone.0113752

**Published:** 2014-11-19

**Authors:** Te-Chuan Chen, Mao-Ling Sung, Hsing-Chun Kuo, Shao-Ju Chien, Chia-Kuang Yen, Cheng-Nan Chen

**Affiliations:** 1 Division of Nephrology, Kaohsiung Chang Gung Memorial Hospital and Chang Gung University College of Medicine, Kaohsiung, Taiwan; 2 Department of Cardiology, St. Martin De Porres Hospital, Chiayi, Taiwan; 3 Institute of Nursing and Department of Nursing, Chang Gung University of Science and Technology, Chronic Diseases and Health Promotion Research Center, CGUST, Taoyuan, Taiwan; 4 Research Center for Industry of Human Ecology, Chang Gung University of Science and Technology, Taoyuan, Taiwan; 5 Division of Pediatric Cardiology, Department of Pediatrics, Kaohsiung Chang Gung Memorial Hospital, Chang Gung University College of Medicine, Kaohsiung, Taiwan; 6 Department of Biochemical Science and Technology, National Chiayi University, Chiayi, Taiwan; Faculty of Biochemistry, Poland

## Abstract

Macrophage accumulation in the arterial wall and smooth muscle cell (SMC) proliferation are features of type 2 diabetes mellitus (DM) and its vascular complications. However, the effects of diabetic monocyte-derived macrophages on vascular SMC proliferation are not clearly understood. In the present study, we investigated the pro-proliferative effect of macrophages isolated from DM patients on vascular SMCs. Macrophage-conditioned media (MCM) were prepared from macrophages isolated from DM patients. DM-MCM treatment induced HASMC proliferation, decreased p21^Cip1^ and p27^Kip1^ expressions, and increased microRNA (miR)-17-5p and miR-221 expressions. Inhibition of either miR-17-5p or miR-221 inhibited DM-MCM-induced cell proliferation. Inhibition of miR-17-5p abolished DM-MCM-induced p21^Cip1^ down-regulation; and inhibition of miR-221 attenuated the DM-MCM-induced p27^Kip1^ down-regulation. Furthermore, blocking assays demonstrated that PDGF-CC in DM-MCM is the major mediators of cell proliferation in SMCs. In conclusion, our present data support the hypothesis that SMC proliferation stimulated by macrophages may play critical roles in vascular complications in DM patients and suggest a new mechanism by which arterial disease is accelerated in diabetes.

## Introduction

Diabetes mellitus (DM) is associated with an increased risk for atherothrombotic complications such as peripheral artery disease, coronary artery disease, and myocardial infarction [1], [2]. It is well documented that macrophage accumulation is a common feature of type 2 diabetes and its complications [3], [4]. Recruitment of monocytes from the peripheral blood to the intima of the vessel wall and the monocytes' subsequent differentiation into macrophages are critical events in atherogenesis. In addition, pathological environments, such as hyperglycemia, may promote macrophage activation and secretion within DM tissues. It has been demonstrated that high-glucose (HG) treatments of human monocytes lead to the increased expression of inflammatory cytokine and chemokine genes [5]. Our previous study also reported that the macrophage inflammatory protein (MIP)-1α and 1β released by HG-treated monocyte-derived macrophages are major mediators for the induction of E-selectin expression in vascular endothelial cells (ECs) [6]. However, the effect of monocyte-derived macrophages isolated from diabetic patients on vascular smooth-muscle-cell (SMC) activation has not been completely understood.

The proliferation and phenotypic changes of vascular SMCs are key events in the development of atherosclerosis and its complications [7]. During the development of atherosclerotic plaque, SMCs migrate from the media into the intimal layer of the arterial wall, where they proliferate and produce an extracellular matrix (ECM), resulting in the formation of intimal hyperplasia [8]. Growth factors, inflammatory cytokines, and chemokines have been implicated as factors that mediate such SMC proliferation [9]. Among these factors, the platelet-derived growth factor (PDGF) family possesses the most potent mitogenic effects for SMC [10]. The G1-to-S phase transit of the cell cycle requires activation of the cyclin-cyclin-dependent kinase (CDK) complex. The cyclin-dependent kinase inhibitors (CKI) such as p21^Cip1^ and p27^Kip1^ have been shown to regulate the activity of these complexes in the G1 phase [11]. p21^Cip1^ and p27^Kip1^ are expressed in quiescent SMCs and are down-regulated following mitogen stimulation [12]. It has been shown that a hyperglycemic environment can activate vascular SMCs and cause vascular dysfunction [13]. In addition, it has also been found that hyperglycemic conditions have mitogenic effects on vascular SMCs [14]. Therefore, examining the proliferative response of vascular SMCs to hyperglycemia may potentially extend our understanding of the pathogenic mechanisms of vascular complications in diabetes.

The functions of SMCs may be modulated by their interaction with other vascular cells such as monocyte-derived macrophages. It has been reported that interactions between monocytes and vascular SMCs may contribute to monocyte retention within the vasculature [15]. Moreover, macrophages can release a wide range of regulatory factors that are growth mediators to affect cell proliferation. Previous studies have indicated that human monocyte-derived macrophages have an inhibitory effect on SMC growth [16], [17]. Evidence also shows that palmitate-stimulated macrophages promote SMC proliferation via the secretion of bone morphogenetic protein (BMP) 2 and BMP4 [18]. Although there is considerable research on the role of macrophages in the development of atherosclerotic lesions, the contribution of macrophages under hyperglycemic conditions to SMC proliferation remains unclear.

SMCs in the media of the artery are encompassed by a network of ECM such as fibrillar collagen. Culturing SMCs on fibrillar collagen promotes the maintenance of the SMC contractile phenotype and exerts an anti-proliferative effect [19]. Since proliferation and accumulation of SMCs are believed to play important roles in the progression of macrophage-rich lesions to fibroatheromas [4], it was hypothesized that monocyte-derived macrophages differentiated from diabetic patients may alter gene expression and affect SMC proliferation. We found that the SMC proliferation induced by macrophages in hyperglycemia is mediated through the up-regulation of microRNA (miR)-17-5p and miR-221. This study presents evidence for a novel mechanism in which miRNAs act synergistically to induce SMC proliferation.

## Methods

### Materials

All culture materials were purchased from Gibco (Grand Island, NY, USA). Rat tail type I collagen was purchased from BD Biosciences (San Diego, CA). Mouse monoclonal antibodies (mAbs) against p21^Cip1^ and rabbit polyclonal antibodies (pAbs) against p27^Kip1^ were purchased from Cell Signaling Technology (Beverly, MA). mAbs against osteopontin (OPN) matrix gla protein (MGP) were purchased from Santa Cruz Biotech (*Santa Cruz*, CA, USA). Other chemicals of reagent grade were obtained from Sigma (St Louis, MO).

### Subjects

The Ethics Committees of St. Martin De Porres Hospital (Chiayi City, Taiwan) approved the study protocol, and written informed consents were obtained from all patients before enrollment. The study group consisted of 13 patients with type 2 diabetes and 9 healthy control subjects. The patients had a mean (±SEM) age of 53.4±8.5 years, body mass index of 31.4±2.5 kg/m^2^, fasting glucose of 185±12.4 mg/dL, triglyceride level of 182.9±16.1 mg/dL, LDL cholesterol level of 94.3±7.8 mg/dL, and hemoglobin A1_C_ of 7.6±1.1%. All patients were treated with glyburide and metformin. None of the patients was primarily insulin dependent. In addition, we recruited 9 volunteers, who were admitted to the St. Martin De Porres Hospital for the purpose of routine physical examinations, as the control subject group. They had a mean (±SEM) age of 49.2±7.9 years, body mass index of 22.7±1.3 kg/m^2^, fasting glucose of 84.6±1.7 mg/dL, triglyceride level of 147.1±8.7 mg/dL, LDL cholesterol level of 95.2±3.8 mg/dL, and hemoglobin A1_C_ of 4.7±0.4%. None of the normal subjects had infectious or inflammatory conditions, or cardiac, renal, or pulmonary decompensated diseases. Volunteers who smoked cigarettes, used alcohol or were under medications (hormonal replacement therapy, nonsteroidal anti-inflammatory drugs, corticosteroids, and anticoagulant drugs) were excluded from this normal subject group.

### Human monocyte isolation

Human monocytes were isolated as previously described [6]. Peripheral blood mononuclear cells (PBMCs) were isolated by Histopaque 1077 density-gradient centrifugation. Monocytes were purified from PBMCs by negative selection using the magnetic-activated cell sorting (MACS) monocyte isolation kit (Miltenyi Biotech, Auburn, CA); PBMCs were first treated with FcγR blocking reagent (human IgG), followed by a hapten/antibody mixture (mixture of hapten-conjugated monoclonal anti-CD3, anti-CD7, anti-CD19, anti-CD45RA, anti-CD56, and anti-IgE antibodies). After treatment with MACS antihapten magnetic microbeads conjugated to monoclonal antihapten antibody, the labeled cells were passed over a MACS column, and the effluent was collected as the negative fraction representing enriched monocytes (>95% purity).

### Preparation of macrophage-conditioned medium (MCM)

Differentiation of monocyte-derived macrophages from diabetes patients or normal control subjects was achieved by culturing the freshly isolated monocytes (5×10^5^ cells/mL) in RPMI-1640 medium supplemented with 10% autologous serum. After 4 days in culture, monocyte-derived macrophages were incubated for another 48 h in fresh serum-free RPMI medium. The conditioned medium was collected, centrifuged, filtered, and defined as diabetic (DM)-MCM and normal control (NC)-MCM.

### Cell culture

Human aortic SMCs (HASMCs) were obtained commercially (Clonetics, Palo Alto, CA) and maintained in F12K medium supplemented with 10% FBS. Cells at passages 3 to 6 were used. Growth of the cells was arrested by incubating in F12K medium with 0.5% FBS for 48 hours before use.

### Collagen matrices

Fibrillar collagen (0.1%) was prepared by mixing 4 mg/ml rat tail type I collagen (25%), 0.1 M NaOH (5%), 2× F12K medium (40%), FBS (10%), and complete medium (F12K with 10% FBS; 20%). The mixture (0.15 ml/cm^2^) was allowed to form fibrillar collagen matrices for at least 1 h at 37°C. SMCs were cultured on the surface of fibrillar collagen as described [19].

### MTT assay and flow cytometric analysis for cell proliferation

Cells were cultured on type I fibrillar collagen in 96-well plates. Cell proliferation was determined by 3-(4,5-dimethylthiazol-2-yl)-2,5-diphenyltetrazolium bromide (MTT) assay. After the incubation period, MTT solution was added to each well to a final concentration of 0.5 mg/mL, and the mixture was incubated at 37°C for 3 hours to allow MTT reduction. The formazan crystals were dissolved by adding dimethylsulfoxide (DMSO) and absorbance was measured at 570 nm with a spectrophotometer.

Cell-cycle distribution was determined by flow cytometry. Cells stained with propidium iodide were analyzed with a FACScalibur (Becton Dickinson), and the data were analyzed by using a mod-fit cell cycle analysis program [19].

### Real-time quantitative PCR

Total RNA preparation and the RT reaction were carried out as described previously [6]. Regular real-time quantitative PCR was performed to confirm PCR array results. PCRs were performed using an ABI Prism 7900HT according to the manufacturer's instructions. Amplification of specific PCR products was detected using the SYBR Green PCR Master Mix (Applied Biosystems). In addition, the designed primers in this study were: p21^Cip1^ forward primer, 5′-CTGAA AGATG GACGC TCAAT-3′; p21^Cip1^ reverse primer, 5′-CGTTT CAGAA GCCAG AAGAG-3′; p27^Kip1^ forward primer, 5′-CTGAA AGATG GACGC TCAAT-3′; p27^Kip1^ reverse primer, 5′-CGTTT CAGAA GCCAG AAGAG-3′; OPN forward primer, 5′- TTGCA GCCTT CTCAG CCAA-3′; OPN reverse primer, 5′- GGAGG CAAAA GCAAA TCACT G-3′; MGP forward primer, 5′- GCTCA ATAGG GAAGC CTGTG AT-3′; MGP reverse primer, 5′- TTTCT TCCCT CAGTC TCATT TGG-3′; 18S rRNA forward primer, 5′-CGGCG ACGAC CCATT CGAAC-3′, 18S rRNA reverse primer, 5′-GAATC GAACC CTGAT TCCCC GTC-3′. Quantification was performed using the 2^−ΔΔCt^ method [6].

### Western blot analysis

SMCs were lysed with a buffer containing 1% NP-40, 0.5% sodium deoxycholate, 0.1% SDS, and a protease inhibitor mixture (PMSF, aprotinin, and sodium orthovanadate). The total cell lysate (50 µg of protein) was separated by SDS-polyacrylamide gel electrophoresis (PAGE) (12% running, 4% stacking) and analyzed by using the designated antibodies and the Western-Light chemiluminescent detection system (Bio-Rad, Hercules, CA), as previously described [19].

### Inhibition of miR-17-5p and miR-221

Anti-miR inhibitors for miR-17-5p, miR-221, and corresponding negative controls were purchased from Ambion, Life Technologies (Austin, TX, USA). The SMCs were then transfected with the miRNA inhibitors or miRNA inhibitor negative control by Oligofectamine Transfection Reagent from Invitrogen, Life Technologies, in accordance with the manufacturer's procedure. The final concentration for miRNA inhibitor was 200 nmol/L. The transfection efficiency of miRNA inhibitors was further verified by Real-time quantitative PCR assay. After transfection with the miR-17-5p and miR-221 inhibitors, the expression levels of miR-17-5p and miR-221 were decreased by 75.3% and 78.7%, respectively.

### ELISA for PDGF-BB and PDGF-CC

The levels of PDGF-BB and PDGF-CC in the MCM were determined by using sandwich ELISA (sensitivity 18 pg/mL; R&D) according to manufacturer's protocols, as previously described [19].

### Statistical analysis

The results are expressed as mean ± standard error of the mean (SEM). Statistical analysis was performed by using an independent Student t-test for two groups of data and analysis of variance (ANOVA) followed by Scheffe's test for multiple comparisons. *P* values less than 0.05 were considered significant.

## Results

### The effect of DM-MCM on SMC proliferation

**Figure 1:**
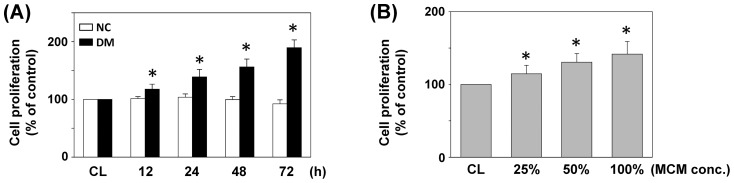
Effect of DM-MCM on cell viability of HASMCs. Bar graphs represent folds of controls (CL) SMCs, mean ± standard error of the mean (SEM) of 5 independent experiments. *P<0.05 versus CL SMCs. (A) SMCs were kept as CL or stimulated with NC-MCM or DM-MCM at the indicated time periods, or (B) stimulated with different concentrations of DM-MCM for 24 h. Cell proliferation was assayed by the MTT test.

The effects of monocyte-derived macrophages from DM patients on cell proliferation were studied by treating SMCs cultured on fibrillar collagen with DM-MCM (vs. NC-MCM). SMCs were stimulated with the two types of MCM at different concentrations for 24 h, or 1× DM-MCM for the times indicated. Cell proliferation was analyzed by MTT assays. As shown in Figure 1A, proliferation of SMC was significantly increased (38%, 56% and 89% after cultivation for 24, 48 and 72 h, respectively) when cultivated with DM-MCM. SMCs cultured with NC-MCM had limited proliferation capacities (Figure 1A). SMCs treated with DM-MCM at different concentrations for 24 h showed a significantly higher growth rate when compared to the control cells (Figure 1B). Table 1 summarizes the flow cytometry analysis of cell distribution in the cell cycle phases. SMCs stimulated with DM-MCM for 24 and 48 h had marked decreases of cells in the G_0_/G_1_ phases and increases in the S-phase (Table 1).

**Table 1:** Cell cycle analysis of SMCs stimulated with NC- and DM-MCM.

		% cells (mean ± SEM)		
	Duration (h)	G_0_/G_1_	S-phase	G_2_/M
**Control**	**0**	87.4±2.1	6.5±0.8	6.1±1.2
**NC-MCM**	**24**	92.3±2.7	5.1±0.6	2.6±1.0
	**48**	92.9±1.9	3.9±0.4	3.2±0.8
	**72**	90.3±2.6	5.6±0.6	4.1±1.1
**DM-MCM**	**24**	72.1±4.8*	19.2±2.4*	8.7±1.4
	**48**	70.6±4.3*	22.3±2.7*	7.1±1.3
	**72**	65.7±6.6*	27.9±4.2*	6.4±0.9

SMCs were kept as controls or on fibrillar collagen with NG- or HG-MCM treatment. Cells were analyzed for DNA content by flow cytometry to show percentages in G_0_/G_1_, synthetic, or G_2_/M phases of cell cycle. Data are mean ± SEM from three independent experiments.

*P<0.05 vs. control cells.

### DM-MCM decreased p21^Cip1^ and p27^Kip1^ and increased synthetic differentiation marker expression in SMCs

**Figure 2:**
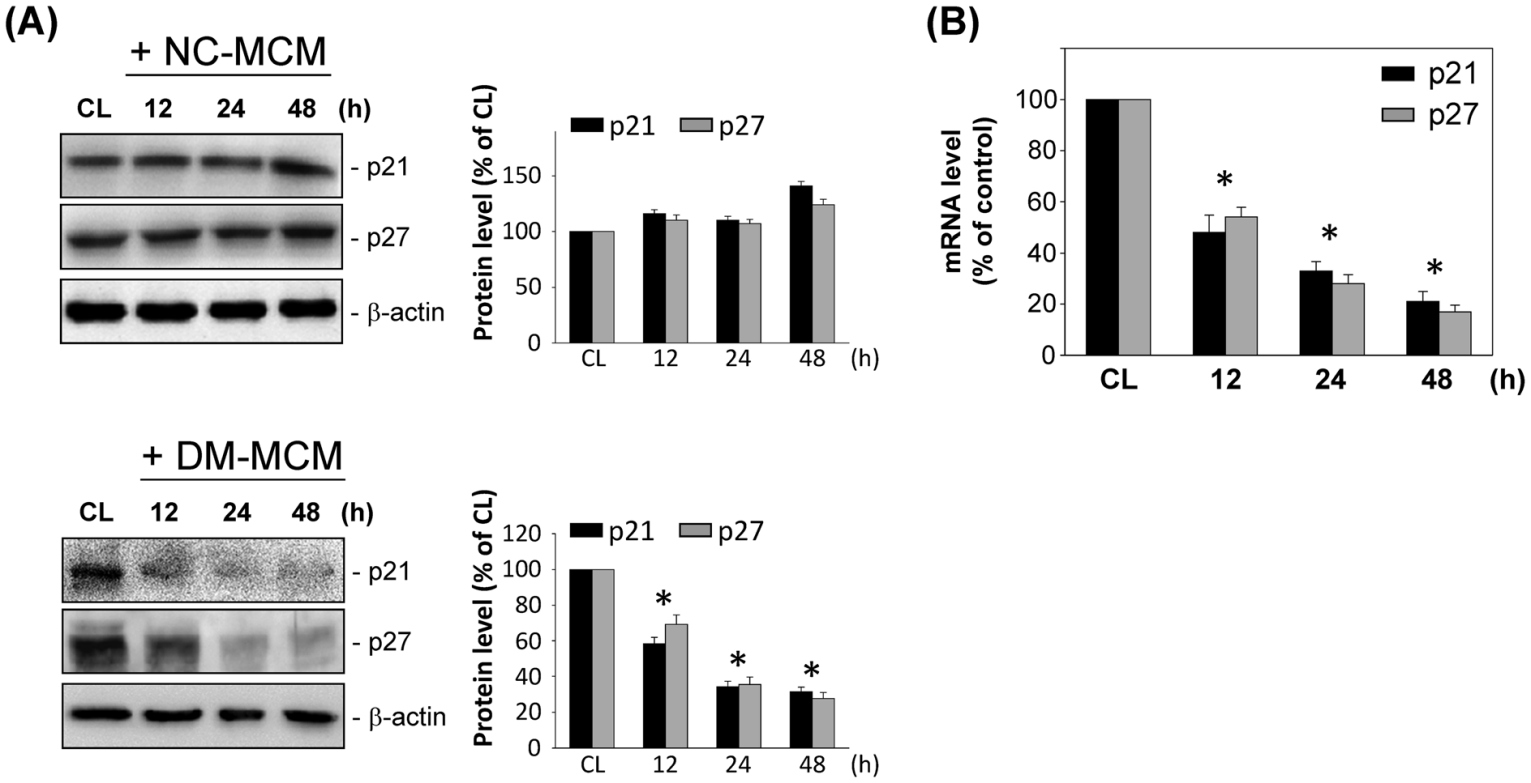
Effects of DM-MCM on p21^Cip1^ and p27^Kip1^ expression. Bar graphs represent folds of CL SMCs, mean ± SEM from 3 independent experiments. *P<0.05 versus CL SMCs. (A) SMCs were kept as CL or stimulated with NC-MCM or DM-MCM for the times indicated. Protein expressions were determined by Western blot analysis. Expression levels of p21^Cip1^ and p27^Kip1^ are presented as band densities (normalized to β-actin) relative to CL. (B) mRNA expressions were determined by real-time PCR analysis and normalized to 18S rRNA.

We examined the effects of DM-MCM on the expression of cell-cycle regulatory proteins p21^Cip1^ and p27^Kip1^. As shown in Figure 2B, SMCs with DM-MCM stimulation decreased the expressions of p21^Cip1^ and p27^Kip1^. However, there was no effect on p21^Cip1^ and p27^Kip1^ expression in SMCs cultured with NC-MCM (Figure 2A). Figure 2C shows that DM-MCM induced decreases in the mRNA expression of p21^Cip1^ and p27^Kip1^ after 12 h of DM-MCM stimulation.

**Figure 3:**
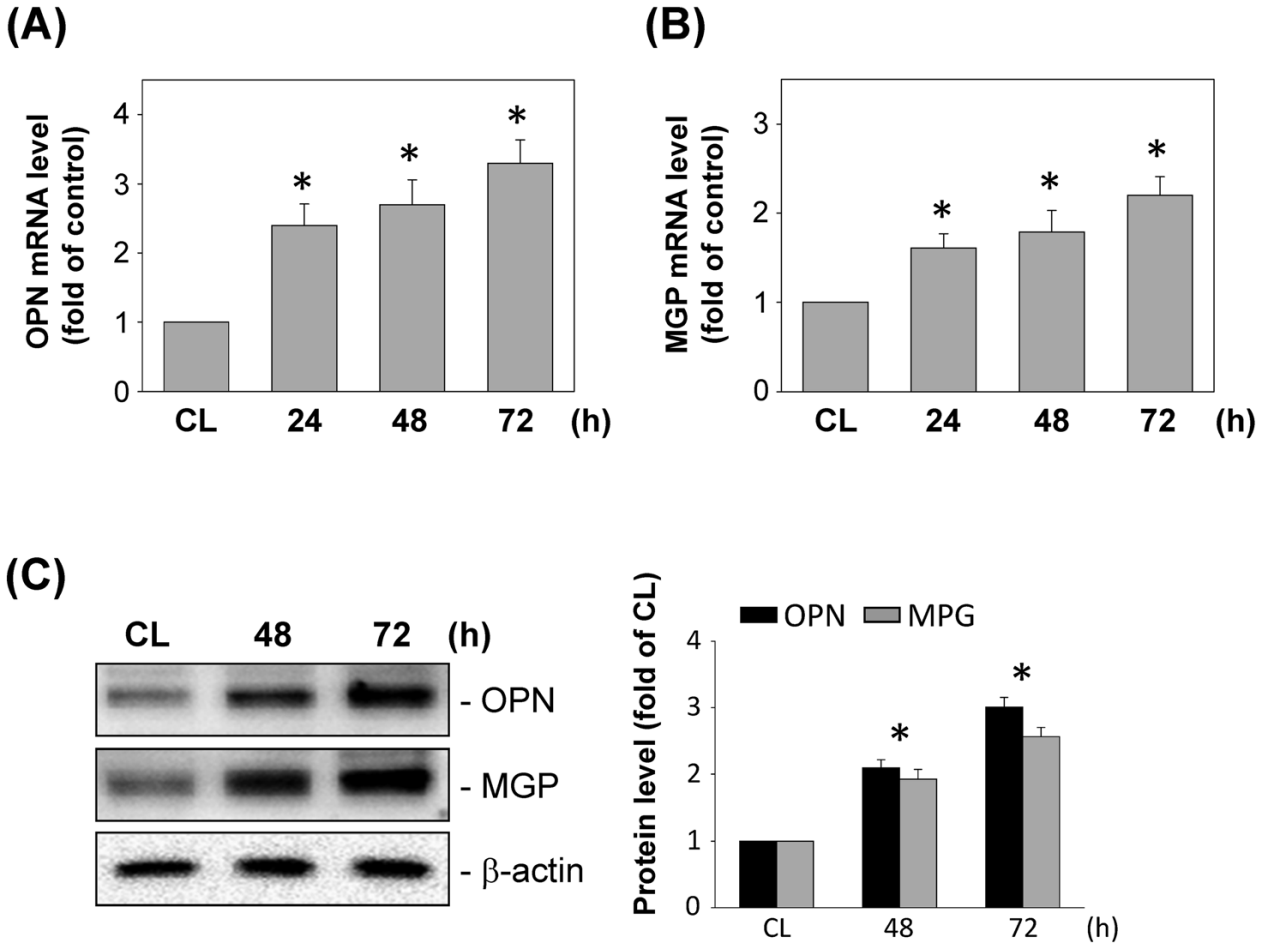
Effects of DM-MCM on synthetic differentiation marker expression in SMCs. Bar graphs represent folds of CL SMCs, mean ± SEM from 3 independent experiments. *P<0.05 versus CL SMCs. (A, B) DM-MCM induced mRNA expressions of osteopontin (OPN) and matrix gla protein (MGP) in SMCs. SMCs were kept as controls (CL) or stimulated with DM-MCM for the times indicated, and the mRNA expressions of OPN (A) and MGP (B) were determined using real-time PCR analysis and normalized to 18S rRNA. (C) SMCs were kept as CL or stimulated with DM-MCM for the times indicated. Protein expressions of OPN and MGP were determined by Western blot analysis. Expression levels of OPN and MGP are presented as band densities (normalized to β-actin) relative to CL.

We also examined the effect of DM-MCM on the expression of synthetic differentiation markers in SMCs. SMCs were cultured on fibrillar collagen, and the mRNA and protein expression of OPN and MGP was analyzed at various time points after cultivation. Real-time PCR analysis indicated the time-dependent increase in the levels of mRNA expression for OPN (Figure 3A) and MGP (Figure 3B) in SMCs on fibrillar collagen. The cultivation of SMCs by DM-MCM also caused significant increases in the OPN and MGP expression at 72 h on fibrillar collagen (Figure 3C).

### miR-17-5p and miR-221 are involved in the regulation of HASMC proliferation

**Figure 4:**
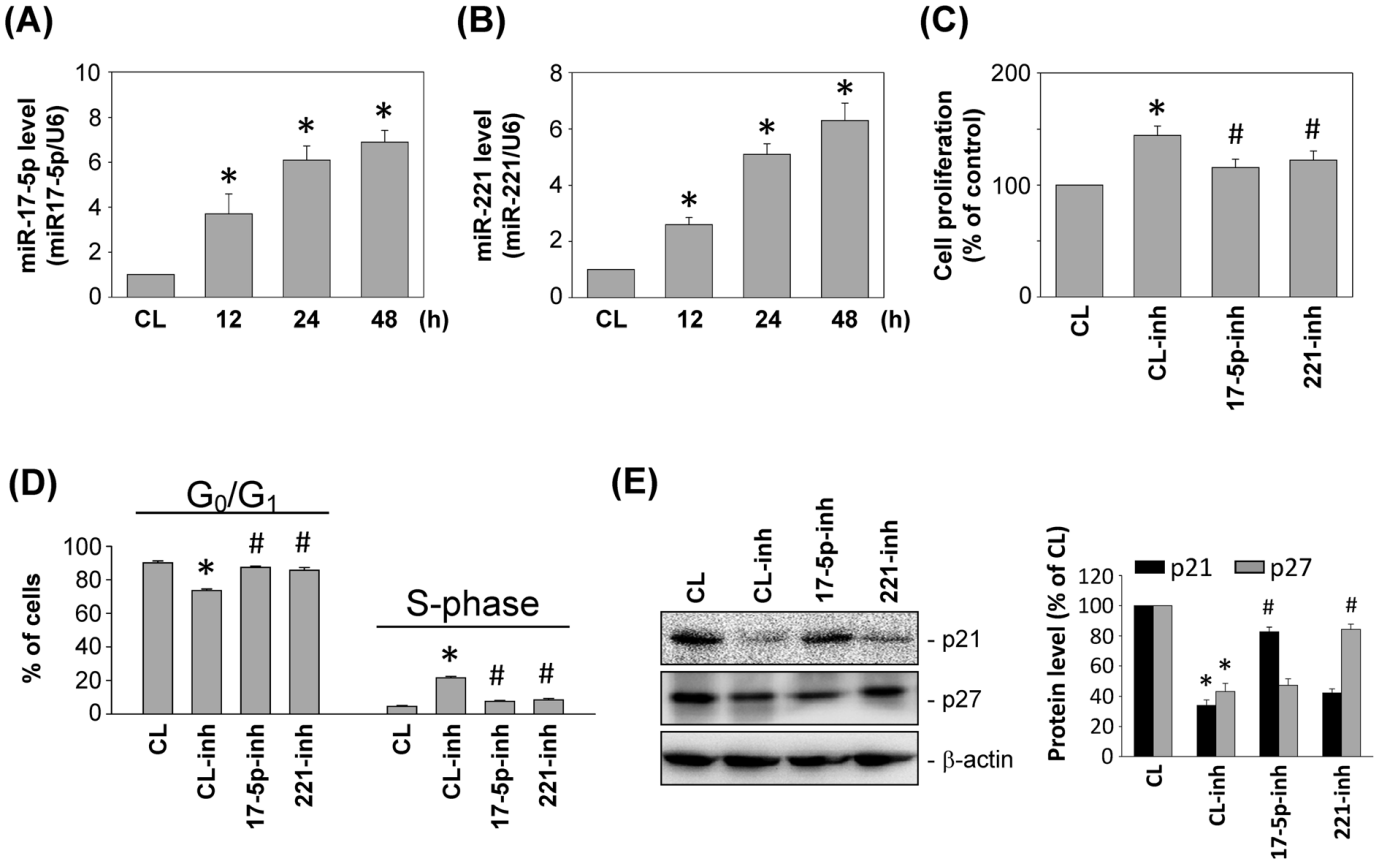
miR-17-5p and miR-221 are involved in the regulation of HASMC proliferation. (A, B) HASMCs were kept as controls (CL) or stimulated with DM-MCM for the times indicated. Relative miR-17-5p (A) and miR-221 (B) levels were determined through real-time PCR in HASMCs and normalized to U6 snRNA from 3 independent experiments. *P<0.05 versus CL SMCs. (C–E) SMCs were kept as CL or pretreated with miR-17-5p inhibitor (17-5p inh), miR-221 inhibitor (221 inh), or miRNA control inhibitor (CL inh) for 24 h, and then stimulated with DM-MCM for 24 h. The results are mean ± SEM from 3 independent experiments. *P<0.05 versus CL. ^#^ *P*<0.05 versus CL inhibitor (CL inh)-treated SMCs with DM-MCM stimulation. Cell proliferation was assayed by the MTT test (C). The distribution of the cell cycle was analyzed by flow cytometry (D). Protein expressions were determined by Western blot analysis. Expression levels of p21^Cip1^ and p27^Kip1^ are presented as band densities (normalized to β-actin) relative to CL (E).

Recent studies have demonstrated that miRNAs are involved in regulating SMC gene expression and proliferation [20]. We therefore examined the effects of DM-MCM on the expression of miRNAs. Stimulation of SMCs with DM-MCM increased the expression of the miR-17-5p (Figure 4A) and miR-221 (Figure 4B), in a time-dependent manner.

To determine whether DM-MCM-induced cell proliferation was mediated by the up-regulation of miR-17-5p and miR-221, SMCs were pretreated with miR-17-5p and miR-221 inhibitors and subsequently stimulated with DM-MCM. The DM-MCM-induced SMC proliferation was significantly inhibited by pretreatments with miR-17-5p and miR-221 inhibitors (Figure 4C). The DM-MCM-induced decrease in the percentage of cells in the G_0_/G_1_ phases and the increase in the percentage of cells in the S-phase were also significantly inhibited by miR-17-5p and miR-221 inhibitors (Figure 4D).

In addition, SMCs were incubated with specific inhibitors of miR-17-5p and miR-221 for 1 h before and during stimulation with DM-MCM, and the expressions of p21^Cip1^ and p27^Kip1^ were analyzed. As shown in Figure 4E, miR-17-5p inhibitor significantly inhibited the DM-MCM-induced expression of p21^Cip1^, whereas miR-221 inhibitor inhibited the DM-MCM-induced expression of p27^Kip1^.

### DM-MCM induced SMC proliferation was mediated by PDGF-CC

**Figure 5:**
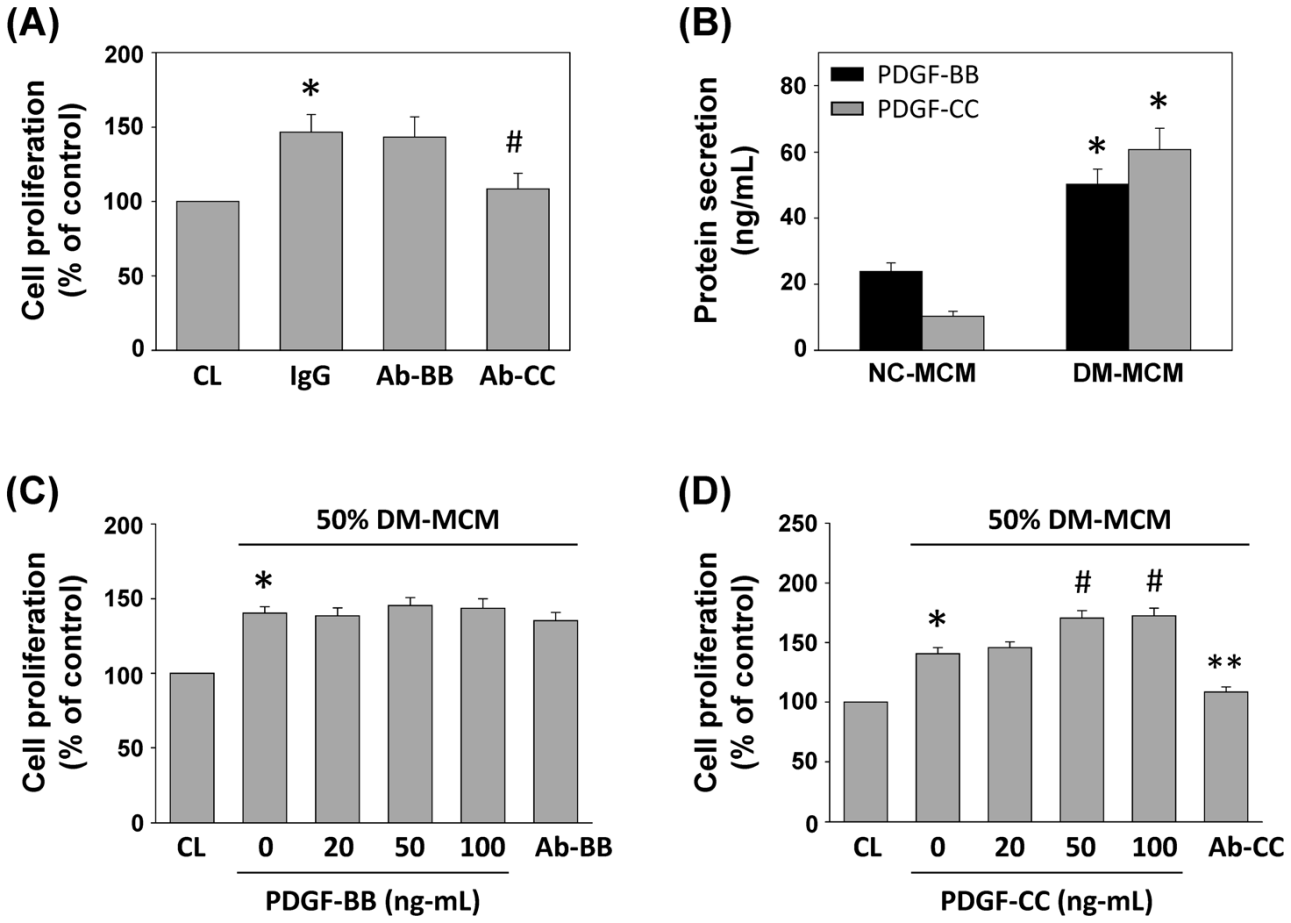
PDGF-CC is the major factor underlying DM-MCM-induced SMC proliferation. (A) Prior to culturing under control conditions (CL) or stimulation with DM-MCM, the DM-MCM and SMCs were pre-incubated with isotype-matched IgG or neutralizing antibodies against PDGF-BB (Ab-BB) or PDGF-CC (Ab-CC) individually for 2 h, and then stimulated with DM-MCM for 24 h. Cell proliferation was assayed by the MTT test from 3 independent experiments. *P<0.05 versus CL. ^#^ *P*<0.05 versus IgG-treated SMCs under DM-MCM stimulation. (B) The expression levels of PDGF-BB and PDGF-CC in MCM were determined by sandwich ELISA from 3 independent experiments. *P<0.05 versus NC-MCM. (C, D) SMCs were kept as CL or stimulated with 50% DM-MCM for 48 h. The proliferation of SMCs was obviously promoted by PDGF-CC at 50 and 100 ng/mL, and the promoting effect was inhibited by the neutralizing antibody against PDGF-CC (Ab-CC) (D). PDGF-BB has no significant effect on promoting SMC proliferation (C). Cell proliferation was assayed by the MTT assay from 3 independent experiments. *P<0.05 versus CL. ^#^ *P*<0.05 versus 50% DM-MCM-treated SMCs. **P<0.05 versus 50% DM-MCM-treated SMCs.

The effect of DM-MCM on SMC proliferation suggests that macrophages under a DM environment may release soluble mediators and exert paracrine effects on SMCs to induce cell proliferation. PDGF was identified in a search for serum factors that stimulate SMC proliferation [10]. As shown in Figure 5A, the incubation of SMCs with PDGF-CC neutralizing antibody, but not PDGF-BB, significantly inhibited DM-MCM-induced SMC proliferation. To confirm these results, the protein levels of PDGF-BB and PDGF-CC in NC- and DM-MCM were analyzed by ELISA. As shown in Figure 5B, culturing of monocyte-derive macrophages caused significant increases of PDGF-BB and PDGF-CC protein secretion in macrophages isolated from diabetic patients.

To further characterize the effect of the PDGF-BB or PDGF-CC in MCM on SMC proliferation and to determine the optimal PDGF concentrations, we examined the responses of SMC to 50% DM-MCM with different concentrations of the PDGF-BB and PDGF-CC (0–100 ng/mL) and their neutralizing antibodies. The results shown in Figure 5D indicated that PDGF-CC promoted SMC proliferation, and the effect was significantly inhibited by neutralizing antibody against PDGF-CC, while PDGF-BB had no significant effect on SMC proliferation (Figure 5C). Figure 5D also indicated that the optimal concentration of PDGF-CC was 50 ng/mL.

## Discussion

Diabetic patients have an increased susceptibility to the development of atherosclerosis [21]. Atherosclerotic lesions in patients with diabetes are characterized by excessive macrophage infiltration, suggesting that the accumulation of monocyte-derived macrophages into the vasculature may be augmented under hyperglycemic conditions [3]. In addition, the migration and proliferation of SMCs are believed to play important roles in the progression of macrophage-rich lesions to fibroatheromas, and diabetic conditions have been shown to enhance this process [22]. It has been suggested that the macrophage-SMC coexistence as neighbors within the vessel walls induces regulatory signals involved in atherogenesis [23]. There is also evidence that hyperglycemia may enhance the interaction of macrophages and SMCs [24]. However, the pathophysiological mechanism of macrophage-SMC interaction remains poorly understood. The present study characterized the roles of two major monocyte-derived macrophage-induced mediators from diabetic patients in the regulation of p21^Cip1^ and p27^Kip1^ expressions by HASMCs. Several lines of evidence from the current study indicate that the effect of macrophages from DM patients on SMC proliferation was mediated by the down-regulation of expressions of p21^Cip1^ and p27^Kip1^ and that this down-regulation was mediated through the differential regulation of the miR-17-5p and miR-221. First, stimulation by DM-MCM induced decreased expression of p21^Cip1^ and p27^Kip1^, as well as increased cell proliferation. Second, stimulation of SMCs by DM-MCM induced expression of miR-17-5p. Pretreatment of the cells with miR-17-5p inhibitor suppressed the DM-MCM-induced down-regulation of p21^Cip1^ and proliferation of the SMCs. Third, stimulation of SMCs with DM-MCM also induced expression of miR-221. Pretreatment of the cells with miR-221 inhibitor decreased the DM-MCM-induced down-regulation of p27^Kip1^ and SMC proliferation.

It is believed that extravasation of macrophages in the arterial wall promotes formation of atherosclerotic lesions. It has also been suggested that diabetes-elicited SMC proliferation occurs secondary to the increased macrophage infiltration into the arterial wall [22]. The effects of HG levels on the proliferative capacity of arterial SMCs are controversial. While several studies have demonstrated that HG levels can stimulate SMC proliferation [14], [25], others have found no stimulatory effect [26], [27]; yet another study has shown an inhibitory effect of HG levels on SMC proliferation [28]. By culturing HASMCs on fibrillar collagen that promotes the maintenance of SMC in a non-proliferative phenotype [19], we have been able to elucidate the factors that signal and control the modulation of HASMC proliferation. The present findings in HASMC cultures showed that treatment with DM-MCM had a stimulatory effect on the proliferation of HASMCs. During cell cycle progression, p21^Cip1^ and p27^Kip1^ have been shown to mediate cell cycle arrest by inhibiting Cdk activities [29]. In this study, mRNA and protein expressions of p21^Cip1^ and p27^Kip1^ were down-regulated in DM-MCM-stimulated HASMCs. Our data also demonstrated that treatment of SMCs with DM-MCM increased the percentage of cells in the S-phase, while the percentage of cells in the G_0_/G_1_ phases decreased.

Several studies have revealed the effects of miRNAs on the modulation of functions of SMCs [20], [30], [31], but the mechanism underlying the DM-MCM-induced proliferation of SMC remains largely unclear. Different miRNAs have been implicated in the regulation of the mitogenic response in SMCs [31], [32]. miR-17-5p is one of the critical miRNAs for cell proliferation [33], and its up-regulation has been shown to modulate p21^Cip1^ expression in cancer cells [34]. miR-221 has also been implicated in the regulation of SMC proliferation and neointimal hyperplasia [35], and it has been reported to play an important role in the regulation of p27^Kip1^ down-regulation in SMCs [36]. The present study demonstrated that DM-MCM stimulates SMC proliferation through at least two miRNAs, as indicated by the DM-MCM-induction of increases in miR-17-5p and miR-221, which may be involved in the mitogenic action. We further showed that the inhibition of miR-17-5p inhibited the DM-MCM-induced down-regulation of p21^Cip1^ but had no effect on p27^Kip1^, while the inhibition of miR-221 affected the down-regulation of p27^Kip1^ but had no effect on p21^Cip1^. It has previously been shown that the differential regulation of cell-cycle regulatory proteins occurs by different mechanisms [37]. On the basis of these studies and our findings, it is reasonable to propose that DM-MCM may stimulate SMC proliferation through the differential regulation of p21^Cip1^ and p27^Kip1^ expressions regulated by different miRNAs.

It has been reported that a variety of regulatory molecules with the ability to regulate SMC or stem cell function in a paracrine fashion are released from macrophages [18], [38]. It is thus postulated that the elicited SMC growth in atherosclerotic lesions in diabetic conditions is due to the secretion of mediators from macrophages, and that macrophages may be directly affected by the diabetic environments. In our previous study, we observed that MIP-1α and 1β are significantly increased only in macrophages after differentiation under an HG environment [6]. In addition, it has been shown that levels of IGF-I in macrophage-rich regions in lesions of atherosclerosis from diabetic pigs were increased compared to non-diabetic controls [39]. Our present data demonstrated that MCM from DM patients significantly enhanced SMC proliferation, and this induction of cell proliferation by DM-MCM was inhibited following the neutralization of the PDGF-CC in DM-MCM. Hence, we suggest that the accumulation of macrophages in the arterial wall thereby increases the PDGF-CC level and contributes to SMC proliferation.

Taken together, our findings contribute new information about the mechanisms by which DM macrophages induce SMC proliferation. Treatment of SMCs with DM-MCM resulted in differential regulation of SMC–proliferation-mediated miRNAs. Activation of the miR-17-5p led to down-regulation of p21^Cip1^, whereas activation of the miR-221 led to decreased expression of p27^Kip1^. These findings provide insights into the mechanisms underlying the interplay between hyperglycemic macrophages with SMCs in modulating SMC function and gene expression, which may well be involved in the development of vascular complications in patients with diabetes.

### Study limitations

There are some inherent limitations to this study. Although this study demonstrated that monocyte-derived macrophages differentiated from diabetic patients may affect SMC proliferation, the overall number of controls and patients was small. This association may therefore be correspondingly under- or over-estimated.
